# Knowledge, dentist confidence and management of periodontal patients among general dentists from Belarus, Lithuania, Macedonia, Moldova and Romania

**DOI:** 10.1186/s12903-020-1033-9

**Published:** 2020-02-10

**Authors:** Jolanta Aleksejūnienė, Alina Pūrienė, Arunas Rimkervicius, Corneliu Amariei, Roxana Oancea, Tatiana Porosencova, Egor Porosencov, Julijana Nikolovska, Elena Mirnaya, Aleksandra Serova-Papakul, Kenneth A. Eaton

**Affiliations:** 1 Faculty of Dentistry, University of British Columbia, Vancouver, Canada; 2 Medical Faculty, University of Vilnius, Vilnius, Lithuania; 3 Romanian Association of OroDental Public Health, Bucharest, Romania; 4 Faculty of Dentistry, University of Medicine and Pharmacy “Victor Babes”, Timișoara, Romania; 5 Faculty of Dentistry, State University of Medicine and Pharmacy “Nicolae Testemițanu”, Chisinau, Republic of Moldova; 6 Faculty of Dental Medicine, University of Sts Cyril and Methodius, Skopje, Macedonia; 7 Dental Faculty, Bellarussian State Medical University, Minsk, Belarus; 8 University College London and University of Kent, Medway, UK

**Keywords:** General dentists, Periodontal patients, Confidence, Patient management

## Abstract

**Background:**

Evidence concerning periodontal practice in Eastern European countries is scarce. The aim of the present study was to investigate periodontal risk knowledge, patient management and self-perceived confidence among General Dentists (GDs) from five Eastern European regarding their provision of periodontal care.

**Methods:**

GDs from Belarus, Lithuania, Macedonia, Moldova and Romania participated in a questionnaire survey. Power calculations were used to identify the sample size for each country. The structured questionnaire included several domains of inquiry. The socio-demographic domain inquired about dentist’s age, gender and years of clinical experience. The dental practice domain inquired about practice location, practising or not practising in a group practice and having or not having a periodontist or a dental hygienist in the practice. The distributions of answers across-countries were compared employing one way ANOVA (comparison of means) or Chi square test (comparison of proportions). For each country, the predictors of the study outcomes: a summative knowledge score for periodontal risks and dentist’s confidence level were identified employing either linear or logistic multiple regression models.

**Results:**

The sample comprised 390 Belarussian, 488 Lithuanian, 349 Macedonian, 316 Moldovan, and 401 Romanian GDs. The majority of GDs (~ 80%) practiced in urban areas. Age and gender distributions differed significantly among countries. Significant across-country differences were found regarding working/not working in a group practice, having/not having access to a periodontist/dental hygienist and in proportions of patients receiving periodontal treatments or being referred to specialists. None of Macedonian patients nor the majority of Moldovan patients (78%) were referred to periodontists. There were also significant across-country differences in diagnosis, patient management and periodontal knowledge. Only in the Lithuanian cohort were dentists’ confidence levels associated significantly with their knowledge. In all countries, taking a medical history was a consistent and significant predictor of having higher periodontal knowledge score. Except in Belarus, periodontal risk assessment was a significantly consistent predictor of certainty levels associated with the provision of periodontal treatments.

**Conclusions:**

There were substantial differences among GDs in the five countries regarding diagnosis, dentist’s confidence and management of periodontal patients.

## Background

Globalization of dentistry has become increasingly important [1, 2]. In order to assure quality patient care and improve oral health globally, uniform standards between countries need to be established and subsequently maintained [3]. In preparation for this standardization and the production of clinical guidelines, it is necessary to understand how dental care is managed in different countries and delivered by different types of dental professionals.

General dentists (GDs) provide multiple treatment modalities for their patients, which treatments they choose is largely related to their practice and patient characteristics [4]. The majority of GDs provide nonsurgical periodontal treatments [5–7]. However, there is a considerable variation in how GDs diagnose and manage periodontal patients [3]. Most importantly, quality patient care should be assured irrespective of whether patients are treated by GDs or periodontists [8]. An accurate diagnosis is an essential first step towards successful dental treatments, but little research is present in this area [9]. GDs usually manage patients with periodontal problems and if available refer individuals to periodontists. Nevertheless, specialist periodontists may not be available, so GDs need the necessary expertise for treating such patients [10, 11]. Consultation between different types of dental professionals is essential in patient care, therefore strong partnerships between GDs and periodontists should be encouraged [12]. However, an overall decrease in periodontal referrals, as well as delayed referrals to specialists have been reported [13, 14]. Comprehensive and timely periodontal care including referrals to specialists is necessary for maintenance of teeth at risk, thus it is important to know how GDs diagnose, treat, counsel and monitor their periodontal patients [15]. Studies in North America have suggested that variation among GDs in periodontal referrals is associated with either diagnostic considerations or treatment patterns [16] and that GDs select their periodontists based on patient satisfaction, successful treatment outcomes and good communication with specialists [17]. No such studies have been performed in Eastern European countries.

The aim of the present study was therefore to investigate periodontal risk knowledge, patient management and self-perceived confidence among GDs from five Eastern European regarding their provision of periodontal care.

## Methods

The present survey, included GDs from Belarus, Lithuania, Macedonia, Moldova and Romania. It took place between 2015 and 2017. The researchers from these countries were members of the Periodontal Epidemiology Special Interest Group of the European Association for Dental Public Health and collaborated in the design of the study. They are all co-authors of this paper. The survey was approved by university ethics committees in each of these countries. They were the Committee of Bioethics of the Republic of Belarus, the Lithuanian Bioethics Ethics Committee, the Ethics Committee of the Macedonian Society of Dental Medicine, the Research Ethics Committee of the State University of Medicine and Pharmacy “Nicolae Testimatanu”, Republic of Moldova, and the Research Ethics committee of the Romanian Association of Orodental Public Health. A sample size calculation, based at a confidence level of 95% and a confidence interval of 0.05, was made using the Australian Statistics Bureau’s sample size calculator [18]. It showed that a random sample of the following numbers of GDs who were active dentists would be representative for the five countries: Belarus (354), Macedonia (328), Moldova (308), Lithuania (349) and Romania (375).

The questionnaire was translated from English into the language of each of the five countries and back-translated into English to check the accuracy of the translations. It was then piloted among randomly selected groups of 10 dentists in each country. A brief explanation of the purpose of the survey was given on the first page of the questionnaire and it was stressed that participation was voluntary and that no individual GDs would be identified in any papers or other communications that resulted from the survey. Distribution of the questionnaire was by email to a random sample of 700 GDs in Belarus and Moldova, 1005 in Lithuania and 1500 GDs in Romania, drawn at random from national dental association member lists from each country. Random sampling from the lists was performed by allocating numbers from 1 upwards to all GDs on the lists and then using a random number generator programme to produce the required number of randomly selected names. In these four countries, reminder emails were sent and the survey was publicised during conferences and continuing education events. In Macedonia, although a list of all dentists was available, it was not possible to obtain their email addresses. To overcome this problem, 20 first year dental students distributed the survey questionnaire in person to a random sample of 700 GDs. The students came from all parts of Macedonia. They distributed the questionnaires in their home areas and were able to visit the clinics where the GDs worked and collected completed questionnaires again in person.

The questionnaire was structured with multiple questions, employing different measurement scales, such as multiple choice questions, Likert scales and visual analogue scales (VAS). A Likert scale with five possible answers from strongly disagree to strongly agree was used, and the VAS measures were on a continuum from one to ten.

The structured questionnaire included several domains of inquiry which were:

- Socio-demographic- with questions about dentist’s age, gender and years of clinical experience.
- Dental practice - with questions about practice location, practising or not practising in a group practice and having or not having a periodontist or a dental hygienist in the practice.
- Diagnosis - with questions about use of radiographs, medical history taking, family and social history and identification of risk factors for periodontal diseases.
- Patient enrollment -with questions about numbers of periodontal patients seen per week and category of clinician (GD, dental hygienist or specialist) providing treatment. Collected information about:
- Patient management - with questions about periodontal maintenance intervals and oral hygiene techniques:

Summative knowledge was scored based on the following series of questions: ‘Which of the following (mark all that apply): smoking, increasing age, hormonal changes in females, AIDS, diabetes, cancer/cancer therapy, medications’ intake, stress and poor oral hygiene do you consider as important risks for the progression of periodontal diseases?’ The response categories to these questions were: yes, no, don’t know. Only correct answers (not incorrect or don’t know) were added together in total risk knowledge score, which had a theoretical range of from zero to nine. Each GD’s confidence level regarding the provision of periodontal treatments was evaluated based on his/her response to the following statement ‘*Being unsure how to treat periodontal patients discourages you from providing such treatments to your patients?’* (Response categories: yes, no, don’t know).

The full questionnaire can be accessed from the Special Interest Working Group for Periodontology page of the European Association for Dental Public Health via the link https://www.eadph.org/download/treatment-of-periodontal-diseases-by-general-dental-practitioners-questionnaire

All statistical analyses were performed employing the IBM SPSS Version 22.0 software. The thresholds for statistical significance in both bivariate and multivariate analyses were set at *p* < 0.05*.* The bivariate analyses included one way ANOVA, with Post hoc Bonferroni adjustment for the comparison of means, and the Chi squared test for comparison of proportions. Two types of multivariate analyses were selected: linear multiple regression models examined multiple predictors for the first study outcome “periodontal risk knowledge score” and the logistic regression tested multiple predictors for the second study outcome “dentist’s confidence level about providing periodontal treatment.”

## Results

A total of 1554 GDs participated. In all five countries the number of responses met or exceeded that required by the power calculation (Table 1). Substantial numbers of GDs, were either uncertain or were unable to define the scientific background/justification for the provision of periodontal treatments to their patients. The highest level of uncertainty was observed in Moldovan GDs and the lowest level of uncertainty was reported among Belarusian and Lithuanian GDs.

**Table 1:** Population, Active Dentists, Periodontal Specialists and Dental Hygienists in the Five Countries surveyed

Country	Population (in millions)	Active Dentists (*)	Participated in survey (**)	Periodontal Specialists	Dental Hygienists
Belarus (2016)	9.7	4478	390 (354)	0	0
Macedonia (2017)	2.1	2240	349 (328)	48	0
Moldova (2016)	3.5	1535	316 (308)	14	17
Lithuania (2017)	2.9	3776	488 (349)	68	261
Romania (2014)	21.3	14,841	401 (375)	259	100

*Dentists resident in the country concerned who are working as dentists or who wish to work as dentists

**Power calculation numbers in brackets

The mean age of Lithuanian and Romanian GDs was significantly lower than that of Belarussian, or Macedonian or Moldovan dentists (*p* < 0.001) (Table 2). Lithuania had the lowest percentage of male GDs (15.4%). The lowest percentage of GDs practising in group practices was found in Macedonia (31.5%). A very low proportion of Moldavian GDs reported access to a periodontist (4.1%) or a dental hygienist (4.5%) in their practice.

**Table 2:** Socio-demographic characteristics of the responding general dentists (GDs)

	Belarus	Lithuania	Macedonia	Moldova	Romania
	mean ± sd	mean ± sd	mean ± sd	mean ± sd	mean ± sd
Age (years)	40.7 ± 11.8	36.6 ± 11.5	41.1 ± 9.5	44.4 ± 10.5	35.7 ± 9.0
Significant differences: Lithuania/Romania vs. Moldova/Macedonia/Belarus(*p* < 0.001)*					
Practice (years)	12.6 ± 11.7	20.0 ± 10.0	13.8 ± 8.9	23.3 ± 7.7	18.1 ± 11.8
Significant differences: Lithuania/Macedonia vs. Moldova/Romania/Belarus (p < 0.001)*					
Gender	N (%)	N (%)	N (%)	N (%)	N (%)
Males	87 (22.3)	75 (15.4)	172 (49.3)	146 (46.2)	148 (36.9)
Females	303 (77.7)	413 (84.6)	177 (50.7)	170 (53.8)	253 (63.1)
Significant across-country differences (p < 0.001)**					
Location of practice					
Urban	370 (94.9)	421 (86.3)	296 (85.1)	249 (78.8)	348 (87.7)
Semi-urban/rural	20 (5.1)	67 (13.7)	44 (12.6)	67 (21.2)	49 (12.3)
Significant differences: Lithuania/Macedonia/Romania/Belarus vs. Moldova (*p* < 0.001)**					
Working in a group practice					
Yes	315 (80.8)	389 (79.7)	110 (31.5)	202 (63.9)	278 (70.4)
No	75 (19.2)	99 (20.3)	239 (68.5)	114 (36.1)	117 (29.6)
Significant across-country differences (p < 0.001)**					
Having a periodontist in your practice#					
Yes	216 (55.4)	144 (29.5)	109 (31.2)	13 (4.1)	43 (11.6)
No	174 (44.6)	344 (70.5)	240 (68.8)	303 (95.9)	292 (78.7)
Significant differences: Lithuania & Macedonia vs. Moldova/Romania/Belarus (*p* < 0.001)**					
Having a dental hygienist in your practice					
Yes	74 (19.0)	279 (57.2)	110 (31.5)	14 (4.4)	26 (7.2)
No	316 (81.0)	209 (42.8)	239 (68.5)	302 (95.6)	334 (92.8)
Significant across-country differences (*p* < 0.001)**					

*Comparison of means using One-way ANOVA with post hoc Bonferroni adjustment

** Comparison of proportions using Chi^2^ test

As far as differences between the countries regarding GDs’ periodontal risk knowledge, their confidence levels about periodontal treatments and different aspects of their patient management were concerned, Lithuanian GDs had significantly better knowledge than either Moldovan or the Macedonian GDs (One-way ANOVA with Post hoc Bonferroni adjustment, *p* < 0.001). Mean knowledge scores (theoretical maximum 9) were as follows: in Belarus 6.1 ± 1.2. Lithuania 6.3 ± 1.0, Macedonia 5.8 ± 1.4, Moldovia 5.7 ± 1.5 and in Romania 5.8 ± 1.4. Significant differences in confidence levels of GDs in the different countries about the provision of periodontal treatments were also found. The highest level of uncertainty among GDs was observed in Moldova and the lowest level of uncertainty was observed in Lithuania (Table 3).

**Table 3:** Periodontal risk knowledge, uncertainty about periodontal treatments and patient enrollment– comparisons among GDs

	Belarus	Lithuania	Macedonia	Moldova	Romania
	mean ± sd	mean ± sd	mean ± sd	mean ± sd	mean ± sd
Knowledge score	6.1 ± 1.2	6.3 ± 1.1	5.8 ± 1.4	5.7 ± 1.4	5.8 + 1.4
Significant differences in means between Lithuania/Belarus& Moldova/Macedonia *					
	N (%)	N (%)	N (%)	N (%)	N (%)
Uncertainty about treatments discourages from providing periodontal treatments^					
Yes	131 (33.6)	88 (18.0)	78 (22.3)	213 (67.4)	154 (45.2)
No	259 (66.4)	326 (66.8)	270 (77.4)	48 (15.2)	171 (50.1)
Don’t know	–	74 (15.2)	1 (0.3)	55 (17.4)	16 (4.7)
Significant across-country proportional differences (*p* < 0.001)**					
Proportion of patients per week requiring periodontal treatments					
none	–	–	–	238 (75.3)	–
1–5	224 (57.4)	257 (52.7)	211 (60.5)	71 (22.5)	291 (75.0)
6–19	166 (42.6)	191 (39.1)	138 (39.5)	7 (2.2)	88 (22.7)
20+	–	40 (8.2)	–	–	9 (2.3)
Significant across-country proportional differences (*p* < 0.001)**					
Proportion of patients per week receiving periodontal treatments (general dentists)					
none	–	–	–	5 (1.6)	261 (68.5)
1–5	145 (37.2)	393 (80.5)	159 (45.6)	306 (96.8)	114 (29.9)
6–19	132 (33.8)	85 (17.4)	108 (30.9)	5 (1.6)	6 (1.6)
20+	113 (29.0)	10 (2.0)	82 (23.5)	–	–
Significant across-country proportional differences (*p* < 0.001)**					
Proportion of patients per week receiving periodontal treatments (dental hygienists)					
none	–	–	–	279 (88.3)	135 (70.3)
1–5	81 (20.8)	227 (46.5)	117 (33.5)	34 (10.8)	21 (11.0)
6–19	204 (52.3)	157 (32.2)	195 (55.9)	3 (0.9)	31 (16.1)
20+	105 (26.9)	104 (21.3)	37 (10.6)	0 (0.0)	5 (2.6)
Significant across-country proportional differences (*p* < 0.001)**					
Proportion of patients per week receiving referrals to periodontists					
none	267 (68.5)	135 (27.7)	349 (100.0)	246 (77.8)	172 (49.9)
1–19	unknown	339 (69.5)	unknown	66 (20.9)	173 (50.1)
20+	unknown	14 (2.9)	unknown	4 (1.3)	–

*Comparison of means using One-way ANOVA with post hoc Bonferroni adjustment

** Comparison of proportions using Chi^2^ test

Bivariate comparisons between countries regarding periodontal patient enrollment and their management showed significant differences among the surveyed countries (Table 3). The lowest proportion of patients requiring periodontal treatments was reported for Moldova, while the highest proportion of enrollment of periodontal patients was reported in Lithuania. In the two countries where there are dental hygienists (Table 1), GDs reported using dental hygienist services less in Romania than in Lithuania. The highest periodontal referral rate was reported in Romania. However, this finding must be treated with caution as incomplete data for this topic were provided by Belarus and Macedonia.

The diagnosis of periodontal diseases, indicated substantial differences between the countries (Table 4). In Belarus, Macedonia and Moldova the majority of GDs reported that they performed full mouth periodontal examinations and selective assessments of pocket depth and attachment loss, while among Lithuanian and Romanian GDs this was not a habitual professional practice. In addition to clinical examination, the majority of Lithuanian GDs reported that they used both periapical and orthopantomograms (OPGs), while GDs in Moldova and Romanian reported taking only OPGs for the diagnosis of periodontal diseases. Only half or less of GDs in countries surveyed provided oral hygiene instructions to all of their patients (Table 5).

**Table 4:** Diagnosis of periodontal diseases – comparisons among GDs

	Belarus n (%)	Lithuania n (%)	Macedonia n (%)	Moldova n (%)	Romania n (%)
Perform full mouth periodontal examinations in all patients					
Yes	238 (61.2)	67 (13.8)	349 (100.0)	8 (2.5)	341 (89.7)
No	152 (38.8)	420 (86.2)	0 (0.0)	308 (97.5)	39 (10.3)
Significant across-country proportional differences (*p* < 0.001)*					
Selective assessments of pocket depth and attachment loss in all patients					
Yes	29 (7.4)	16 (3.3)	196 (56.2)	10 (3.2)	15 (3.9)
No	361 (92.6)	472 (96.7)	153 (43.8)	306 (96.8)	370 (96.1)
Significant differences: Macedonia vs. other participating countries (*p* < 0.001)*					
Perform risk assessment for periodontal diseases					
Yes	381 (97.7)	215 (44.1)	319 (91.4)	180 (57.0)	243 (63.1)
No	9 (2.3)	273 (55.9)	30 (8.6)	136 (43.0)	142 (36.9)
Significant across-country differences (*p* < 0.001)*					
Periapical radiographs taken for the diagnosis of periodontal diseases					
Yes		303 (62.1)	227 (65.0)	50 (15.8)	154 (42.8)
No		185 (37.9)	122 (35.0)	266 (84.2)	206 (57.2)
Significant across-country proportional differences (*p* < 0.001)*					
Orthopantomograms taken for the diagnosis of periodontal diseases					
Yes		351 (71.9)	82 (23.5)	260 (82.3)	312 (86.7)
No		137 (28.1)	267 (76.5)	56 (17.7)	64 (13.3)

Significant across-country proportional differences (*p* < 0.001)*

* Chi^2^ test

**Table 5:** Management of periodontal diseases - comparisons among GDs*

Provide periodontal maintenance at regular follow-up visits					
Yes	310 (79.5)	415 (85.0)	286 (81.9)	261 (82.6)	296 (79.4)
No	80 (20.5)	73 (15.0)	93 (18.1)	55 (17.4)	61 (16.4)
Significant across-country proportional differences (*p* < 0.001)					
Provide oral hygiene instructions to all patients					
Yes	196 (50.3)	163 (33.4)	7 (2.0)	111 (35.1)	150 (38.6)
No	114 (49.7)	325 (66.6)	342 (98.0)	205 (64.9)	239 (61.4)
Significant across-country proportional differences (*p* < 0.001)					
Educate all patients about dental flossing					
Yes	43 (11.0)	113 (23.2)	232 (66.5)	31 (9.8)	92 (23.7)
No	347 (89.0)	375 (76.8)	117 (33.5)	285 (91.2)	296 (76.3)
Significant across-country differences (*p* < 0.001)					
Teach all patients how to use interdental brushes					
Yes	140 (35.9)	69 (14.1)	191 (54.7)	21 (6.6)	80 (20.5)
No	250 (64.1)	419 (85.9)	158 (45.3)	295 (93.4)	311 (79.5)

Significant across-country proportional differences (*p* < 0.001)

* Chi^2^ test

The overall trend in multivariate analyses (Tables 6 and 7) was that the proportion of explained variance by a set of predictors was relatively low and that only a few predictors were significant in regression models. A larger number of significant predictors was found for the outcome ‘periodontal risk knowledge’ than for the outcome ‘the GDs’ confidence regarding the provision of periodontal treatments’. Varying predictors for the ‘periodontal risk knowledge’ were found in: Belarus (‘taking full medical history including medication use’, ‘periodontal risk assessment’, Lithuania (‘practice location’, ‘working in a group practice’, ‘clinical experience’), Macedonia (‘taking full medical history including medication use’), Romania (‘taking radiographs for the PD diagnosis’, taking family history of periodontal diseases’) and Moldova (‘taking radiographs for the PD diagnosis’). Except for Belarus, the same significant predictor “assessment of periodontal risks” for the outcome “confidence in provision of periodontal treatments” was found in regression models tested separately for Lithuania, Macedonia and Moldova.

**Table 6:** Predictors of knowledge comparisons among GDs *

	Belarus		Lithuania		Macedonia		Moldova		Romania	
PREDICTORS	Adj. R^2^ = 0.041		Adj. R^2^ = 0.097		Adj. R^2^ = 0.092		Adj. R^2^ = 0.067		Adj. R^2^ = 0.030	
	β coeff.	*p*	β coeff.	*p*	β coeff.	*p*	β coeff.	*p*	β coeff.	*p*
Practice location	0.031	0.558	0.098	0.028	0.011	0.836	0.049	0.386	0.029	0.587
Working in a group practice	0.003	0.951	0.124	0.006	0.011	0.835	0.048	0.405	0.016	0.543
Clinical experience > 5 years	0.066	0.190	0.135	0.004	0.024	0.645	0.038	0.503	0.002	0.967
Radiography for periodontal diagnosis	0.054	0.281	0.012	0.905	0.065	0.216	0.215	< 0.001	0.141	0.011
Medical history including medications	0.165	0.001	0.083	0.082	0.283	< 0.001	0.132	0.042	0.021	0.726
Family history of periodontal diseases	0.007	0.985	0.079	0.131	0.098	0.719	0.046	0.473	0.158	0.006
Periodontal risk assessment	0.098	0.050	0.054	0.276	0.221	0.036	0.065	0.301	0.085	0.134

*Linear Multiple Regression Models

**Table 7:** Predictors of uncertainty comparisons among GDs *

PREDICTORS	Nagelkarke R^2^ = 0.038		Nagelkarke R^2^ = 0.067		Nagelkarke R^2^ = 0.051		Nagelkarke R^2^ = 0.067		Nagelkarke R^2^ = 0.052	
	OR	*p*	OR	*p* value	OR	*p*	OR	*p* value	OR	*p*
Practice location	1.2	0.648	1.4	0.190	1.4	0.247	1.4	0.432	1.1	0.811
Working in a group practice	0.6	0.112	0.8	0.408	0.7	0.264	1.6	0.176	0.8	0.258
Clinical experience > 5 years	0.6	0.145	1.4	0.172	0.8	0.511	0.2	0.123	0.8	0.369
Radiographs for periodontal diagnosis	0.5	0.028	1.4	0.144	1.4	0.409	1.4	0.451	0.9	0.646
Medical history including medications	0.9	0.940	1.5	0.062	1.1	0.878	0.8	0.614	1.7	0.076
Family history of periodontal diseases	1.3	0.531	0.9	0.562	1.0	0.987	1.4	0.428	0.7	0.110
Periodontal risk assessment	0.6	0.672	2.4	0.001	0.3	0.009	0.1	0.305	2.0	0.007

*All multivariate regression models were significant (*p* < 0.01). OR = Odds Ratio

## Discussion

The present study examined periodontal risk knowledge, GD’s confidence levels regarding the diagnosis, patient enrollment and management among general dentists in five Eastern European countries. The majority of GDs (~ 80%) in these countries practiced in urban areas. Multiple significant and substantial differences between the countries were found in the enrollment, management and referral of periodontal patients. There were also substantial differences in how dental practices were set up, such as working or not working in a group practice, or having or not having a periodontal specialist or dental hygienist available. The most pronounced differences were related to proportions of patients receiving periodontal treatments or being referred to specialists. Unsurprisingly, as there are no specialist periodontists in Belarus, respondents answered that none of their patients were referred to periodontists. In addition, there were significant differences between the countries in GDs’ confidence levels and periodontal knowledge. The level of confidence was associated with knowledge only among Lithuanian general dentists.

Although periodontal probing is considered a gold standard for periodontal diagnosis, probing was not employed for all patients. This finding is in accordance to a previous study reporting a negative relationship between clinical experience and the frequency of probing [5].

The findings from the five Eastern European countries indicate a necessity to establish universal and standardized clinical guidelines for the periodontal care in these countries. Moreover, the referral rates among GDs in the countries, which took part in this survey, were relatively low, indicating that strong partnerships between GDs and specialists have not been established. However, in part this may reflect a lack of, or very small number of periodontal specialists in some of the countries. In order to maintain patient trust and provide quality dental care for all patients, ethical implications, inherent in the relationship between GDs and specialists need to be considered [19]. Unhealthy competition between GDs and periodontists to hold on to their patients should be discouraged because a substantial number of new patients for specialists can be generated from GDs. Conversely, specialists can be a source of patients for GDs [20]. This may have been a factor in Macedonia where there are four dental schools for a population of just over two million and in Romania where a number of dentists are unemployed or underemployed [21].

The limitations of the present study need to be acknowledged. Only basic enquiries about patient enrollment and patient management were made. Overall, although the sample sizes met those indicated by the power calculations there can be some uncertainty that the GDs who responded to the survey were in fact typical as they had the enthusiasm to complete the questionnaire and also whether or not the lists provided by the national dental associations included the email addresses of all GDs. However, the mean age and gender distribution of respondents in all five countries were virtually the same as those of those for all dentists in these countries. As mentioned previously, the method for distributing the questionnaire was different in one of the countries (Macedonia) and this could be considered a weakness. However, the use of first year dental students to distribute and collect completed questionnaires enabled data gathering for the survey to be completed in a fortnight and the students concerned visited dental clinics in all parts of their country and were pleased to be involved in research at an early stage of their studies. The study did not collect in depth information about specificity of patient care. These aforementioned limitations preclude examining patterns of multiple associations and how these associate with care provided to periodontal patients. Another potential limitation that there is always uncertainty about the validity of some answers obtained through self-reports. An example of this relates to the reports from Belarus, where in spite of the fact that there are no officially recognised periodontal specialists nor dental hygienists (Table 1), 216 (55.4%) of the respondents reported that they had a periodontist in their practice and 74 (19%) that they had a dental hygienist (Table 2). When this anomaly was queried with the colleague who performed the study in Belarus, it was explained that the respondents may have taken the term periodontist to mean someone with an interest in periodontology, rather than someone who had undergone full postgraduate training in periodontology and was a registered periodontal specialist. As far as dental hygienists were concerned, due to a lack of understanding of the profession of dental hygienist, some of the Belarusian respondents may have interpreted this as meaning a clinician who provided dental prophylaxes and gave oral hygiene advice.

Thus the findings of the present study can only serve as the first step in information collection towards the preparation of uniform standardized requirements for the periodontal care. Furthermore, the study did not consider the influence of payment systems on the provision of treatment. It is necessary to investigate if the differences between the five countries that took part in this study are also found in other European and other countries worldwide. The Periodontal Epidemiology Special Interest Group of the European Association of Dental Public Health is taking this issue forwards and it would be helpful do so in collaboration with national and continental periodontal associations.

In summary, a substantial variation among GDs from five Eastern European countries was found with regards to multiple aspects of periodontal patient care, in addition to a lack of simple knowledge about periodontal risks, at least in some GDs. The importance of lifelong learning as an important requirement of professional performance needs to be emphasised. Another important consideration is because dentists belong to a self-regulated medical profession, accurate self-assessment of one’s clinical performance is of key importance [22].

Therefore, GDs need to upgrade their knowledge and awareness in all aspects of contemporary dentistry including periodontology regularly [5]. National periodontal societies in Europe and the European Federation of Periodontology can and should play a key role in improving periodontal knowledge. Improvement of knowledge among GDs and their lifelong learning can be achieved in several ways. Continuous professional development can be facilitated by collaborating closely with local specialists [12]. Publications such as the British Society of Periodontology’s “Good Practitioners Guide to Periodontology” [23] can help to facilitate this process**.** Within dental schools, the International Federation for Dental Educators and Associations http://www.IFDEA.org can serve as a professional platform for exchange of knowledge and expertise between different types of dental professionals^1.^ Similarly, the Association for Dental Education in Europe (ADEE) provides guidance for the Dental Education Quality Assurance across the European Higher Education Area and a series of resources from which dental schools can choose the ones that are most appropriate for their needs [24]. Another approach to enhance global standardisation could be to establish computer-assisted e-learning in the training of dentists and the dental team in the future [25]. The future standardisation of clinical periodontology should reflect the principles of prevention of disease, specificity to individual patients, active patient participation and achieve predicted outcomes [26].

## Conclusions

Substantial differences among GDs from five Eastern European countries regarding dentists’ confidence levels, periodontal diagnosis, patient management including their referral to specialists were observed. There is a need to perform this survey in a wider range of countries.

## Data Availability

Once published, the data and material in this paper will be available on open access. Data from each country can be obtained from the authors from the country concerned.

## Abbreviations

AIDS: Acquired Immune Deficiency Syndrome
ANOVA: Analysis of Varience
GDs: General Dentists
IBM SPSS: International Business Machines Statistical Package for Social Sciences
PD: Periodontal
VAS: Visual Analogue Scale
